# Supplementary material for: Transcriptomic Insights into Mechanisms of Early Seed Maturation in the Garden Pea (Pisum sativum L.)
Source: Cells. 2020 Mar 23;9(3):779. doi: 10.3390/cells9030779 (PMC7140803; doi:10.3390/cells9030779)

Zhongwan−6,25(3)

Global Summary

%DE = 0.24

genes with fdr < 0.2 = 461 (214 + / 247 −)

genes with fdr < 0.1 = 413 (200 + / 213 −)

genes with fdr < 0.05 = 396 (194 + / 202 −)

genes with fdr < 0.01 = 346 (172 + / 174 −)

genes in genesets = 0

<FC> = 0

<t−score> = 0

<p−value> = 0.01

<fdr> = 0.76

Global Genelist

Rank	ID	log(FC)	p−value	fdr	Metagene	Description
1	TRINITY_DN2	−0.55	2e−16	3e−15	1 x 1	
2	TRINITY_DN9	1.77	2e−16	3e−15	30 x 30	
3	TRINITY_DN7	−0.62	2e−16	3e−15	30 x 1	
4	TRINITY_DN7	0.87	2e−16	3e−15	30 x 30	
5	TRINITY_DN5	2.34	2e−16	3e−15	30 x 30	
6	TRINITY_DN5	0.83	2e−16	3e−15	1 x 1	
7	TRINITY_DN5	−0.44	2e−16	3e−15	1 x 30	
8	TRINITY_DN5	0.49	2e−16	3e−15	1 x 23	
9	TRINITY_DN7	−1.13	2e−16	3e−15	1 x 1	
10	TRINITY_DN5	0.46	2e−16	3e−15	30 x 4	
11	TRINITY_DN4	−2.79	2e−16	3e−15	1 x 1	
12	TRINITY_DN9	−0.48	2e−16	3e−15	4 x 30	
13	TRINITY_DN9	−0.83	2e−16	3e−15	30 x 1	
14	TRINITY_DN2	0.53	2e−16	3e−15	29 x 30	
15	TRINITY_DN2	−0.67	2e−16	3e−15	1 x 1	
16	TRINITY_DN2	−0.74	2e−16	3e−15	30 x 1	
17	TRINITY_DN2	1.38	2e−16	3e−15	30 x 30	
18	TRINITY_DN2	−0.58	2e−16	3e−15	1 x 30	
19	TRINITY_DN2	−0.75	2e−16	3e−15	1 x 1	
20	TRINITY_DN2	0.5	2e−16	3e−15	1 x 1	

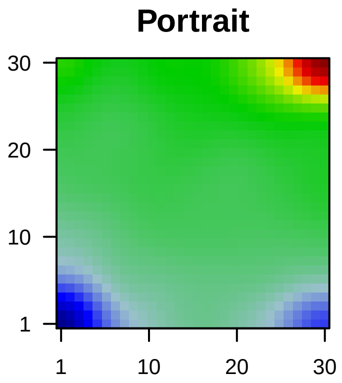

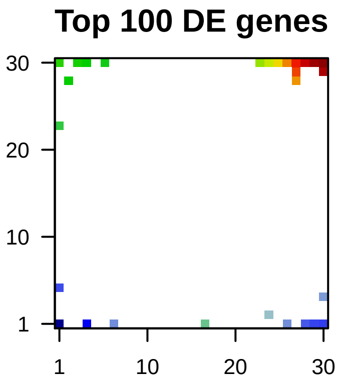

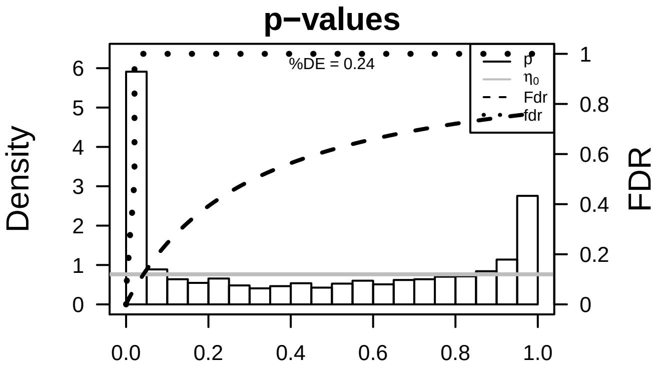

Supplement: Supplementary file 1 [file cells-09-00779-s001.zip › Supplementary materials/FileS4.zip!/FileS3/TFs - Results/Summary Sheets - Samples/Zhongwan.6.25.3..pdf]
